# TGF-β Signaling Controls Embryo Development in the Parasitic Flatworm Schistosoma mansoni

**DOI:** 10.1371/journal.ppat.0030052

**Published:** 2007-04-06

**Authors:** Tori C Freitas, Euihye Jung, Edward J Pearce

**Affiliations:** Department of Pathobiology, School of Veterinary Medicine, University of Pennsylvania, Philadelphia, Pennsylvania, United States of America; University of Edinburgh, United Kingdom

## Abstract

Over 200 million people have, and another 600 million are at risk of contracting, schistosomiasis, one of the major neglected tropical diseases. Transmission of this infection, which is caused by helminth parasites of the genus *Schistosoma,* depends upon the release of parasite eggs from the human host. However, approximately 50% of eggs produced by schistosomes fail to reach the external environment, but instead become trapped in host tissues where pathological changes caused by the immune responses to secreted egg antigens precipitate disease. Despite the central importance of egg production in transmission and disease, relatively little is understood of the molecular processes underlying the development of this key life stage in schistosomes. Here, we describe a novel parasite-encoded TGF-β superfamily member, *Schistosoma mansoni Inhibin/Activin (SmInAct),* which is key to this process. In situ hybridization localizes *SmInAct* expression to the reproductive tissues of the adult female, and real-time RT-PCR analyses indicate that *SmInAct* is abundantly expressed in ovipositing females and the eggs they produce. Based on real-time RT-PCR analyses, *SmInAct* transcription continues, albeit at a reduced level, both in adult worms isolated from single-sex infections, where reproduction is absent, and in parasites from IL-7R^−/−^ mice, in which viable egg production is severely compromised. Nevertheless, Western analyses demonstrate that SmInAct protein is undetectable in parasites from single-sex infections and from infections of IL-7R^−/−^ mice, suggesting that SmInAct expression is tightly linked to the reproductive potential of the worms. A crucial role for SmInAct in successful embryogenesis is indicated by the finding that RNA interference–mediated knockdown of *SmInAct* expression in eggs aborts their development. Our results demonstrate that TGF-β signaling plays a major role in the embryogenesis of a metazoan parasite, and have implications for the development of new strategies for the treatment and prevention of an important and neglected human disease.

## Author Summary

Schistosomes are parasitic worms that infect hundreds of millions of people in developing countries. They cause disease by virtue of the fact that the eggs that they produce, which are intended for release from the host in order to allow transmission of infection, can become trapped in target organs such as the liver, where they induce damaging inflammation. Egg production by female schistosomes is critically dependent on the presence of male parasites, without which females never fully develop, and (counterintuitively) on the contribution of signals from the host's immune system. Very little is understood about the molecular basis of these interactions. Here, we describe a newly discovered schistosome gene, which is expressed in the reproductive tract of the female parasite and in parasite eggs. The protein encoded by this gene is made only when females are paired with males in an immunologically competent setting. Using recently developed tools that allow gene function to be inhibited in schistosomes, we show that the product of this gene plays a crucial role in egg development. Examining how the expression of this gene is controlled has the potential to provide insight into the molecular nature of the interactions between male and female parasites and their hosts. Moreover, the pivotal role of this gene in the egg makes it a potential target for blocking transmission and disease development.

## Introduction

Amongst the Bilateria, transforming growth factor–β (TGF-β) signaling is recognized as playing an essential role in embryogenesis in deuterostomes and in arthropod protostomes, but its role in lophotrochozoan protostomes is unclear [1]. Schistosomes, the causative agents of schistosomiasis, one of the major neglected tropical diseases [2,3], are metazoan parasites that belong to the lophotrochozoan phylum Platyhelminthes.

Components of TGF-β signaling have been molecularly characterized in metazoans throughout the animal kingdom. Activation of this pathway begins at the cell surface when a dimeric ligand binds a complex consisting of types I and II receptor serine/threonine kinases [4]. Upon ligand binding, the constitutively active type II receptor phosphorylates and activates the type I receptor, which then phosphorylates cytoplasmic Smad proteins that translocate to the nucleus, where they mediate gene expression [4]. Components of a functional TGF-β pathway(s), including one type I receptor [5] (Schistosoma mansoni receptor kinase-1 [SmRK1], S. mansoni transforming growth factor–β type I receptor [SmTβ RI]), one type II receptor [6,7] (SmRK2, SmTβ RII), and three Smads [8–10], have been identified in *S. mansoni,* with nearly all components localized to either the surface of the worm or reproductive tissues of the female [5–9,11]. Nevertheless, while nearly the entire transcriptome of S. mansoni has been examined with the identification of 163,000 expressed sequence tags (ESTs) [12], a ligand of parasite origin for the TGF-β pathway(s) has remained elusive. This has led to the hypothesis that the ligands for schistosome TGF-β receptors are of host origin [5,13,14], and a suggestion that host TGF-β, signaling through SmRK2, plays a role in the pairing of male and female parasites [7].

Sexually mature S. mansoni live within the mesenteric vasculature, where each female produces approximately 300 eggs each day. Transmission of schistosomiasis depends upon the release of parasite eggs from the human host. Development of an immature egg into a mature egg containing a miracidium, the stage of the parasite that invades the intermediate fresh water snail host, occurs outside of the female worm, and takes approximately 5 d. Many of the eggs produced by schistosomes fail to reach the external environment, but instead become trapped in host tissues, where pathological changes caused by the immune responses to secreted egg antigens cause disease [15]. Despite the central importance of egg production in transmission and disease, and recent advances in proteomics and transcriptomics [12,16–18], essentially nothing is known of the molecular pathways involved in embryogenesis in schistosomes.

In this study, we describe the cloning and characterization of a S. mansoni TGF-β homolog, *S. mansoni Inhibin/Activin (SmInAct)*. Although we found *SmInAct* to be expressed in adult male and female parasites, and in eggs, the localization of *SmInAct* expression to the reproductive organs of female parasites focused our attention on the role of this gene in egg production. A role for *SmInAct* in reproduction was supported by analyses of female parasites recovered from infertile infections, in which we found that SmInAct protein was undetectable. Confirmation of the importance of this TGF-β superfamily member in the reproductive process was obtained from RNA interference (RNAi) studies, in which targeted knockdown of *SmInAct* in female worms or directly in the eggs that they produce resulted in a marked cessation of embryogenesis.

## Results

### Cloning and Sequence Analysis of *SmInAct*

**Figure 1:**
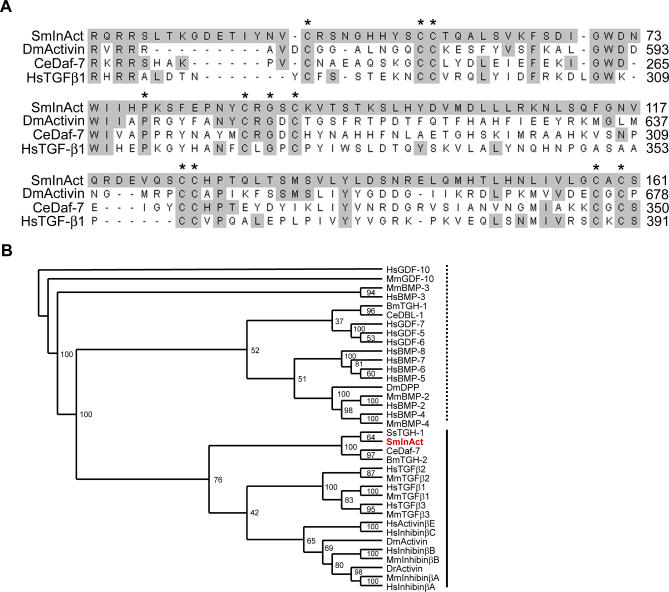
SmInAct Is a Member of the TGF-β/Activin Subfamily (A) ClustalW alignment of the C-terminal domain of the SmInAct protein with three other members of the TGF-β/Activin subfamily. Amino acids identical to the SmInAct sequence are shaded gray. Numbers to the right indicate position of the last amino acid in the row within each respective full-length sequence. Stars indicate invariant amino acid residues in TGF-β homologs. (B) Phylogenetic dendrogram demonstrating that SmInAct is a member of the TGF-β superfamily. SmInAct (red) is shown clustering among members of the TGF-β/Activin subfamily (solid line), and not with members of the BMP/growth differentiation factor subfamily (dashed line). Conserved residues in the C-terminal region of each homolog (final 94–106 amino acids) were used in the analysis. Percentages at branch points are based on 1,000 bootstrap runs.

*SmInAct* was identified through a *t*blast*n* search of the Wellcome Trust's Sanger Institute's S. mansoni genome sequence using the C-terminal region of the Drosophila melanogaster dActivin sequence. We were unable to identify SmInAct in EST databases regardless of whether we searched using the coding or 3′–untranslated region (UTR) sequences. The 5′ and 3′ ends of SmInAct were amplified via rapid amplification of cDNA ends (RACE) using primers designed from within putative coding sequence and adult S. mansoni cDNA as template. The 1.3-kb, full-length *SmInAct* transcript contains 10 base pairs (bp) of 5′UTR, 808 bp of 3′UTR, and a poly-A tail. The deduced amino acid sequence of SmInAct is 161 residues long and contains many of the molecular hallmarks for a TGF-β, including a putative basic proteolytic cleavage site located at position 32 as RQRR where the bioactive, C-terminal domain (126 amino acids) is enzymatically separated from the N-terminal pro-domain. Nine invariant cysteine moieties, and invariant proline and glycine residues (Figure 1A) essential for the proper dimerization and tertiary structure of a TGF-β homolog, are all predicted in SmInAct. The deduced amino acid sequence of SmInAct contains one putative N-linked glycosylation site at position 110. Within the bioactive domain, SmInAct is 27% identical to both DAF-7 from Caenorhabditis elegans and dActivin from *D. melanogaster,* and 29% identical to human TGF-β 1 (Figure 1A). Phylogenetic analysis of SmInAct among other TGF-β superfamily members groups this homolog with members of the TGF-β/Activin subfamily (Figure 1B), and further clusters SmInAct phylogenetically with TGF-β homologs from the free-living nematode C. elegans (DAF-7) and the parasitic nematodes Brugia malayi (Bm-TGH-2) and Strongyloides stercoralis (Ss-TGH-1).

### SmInAct Transcript and Protein Expression and Localization

**Figure 2:**
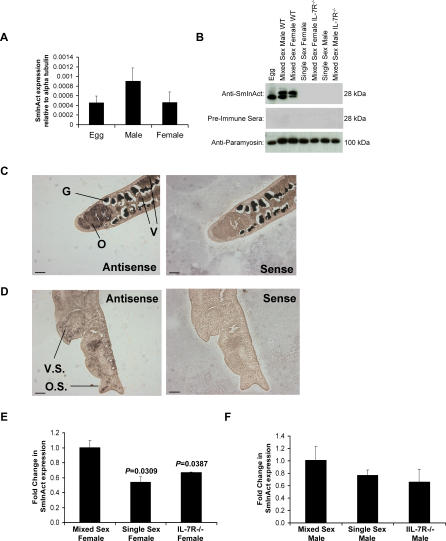
SmInAct Expression Is Linked to Reproductive Capability (A) *SmInAct* is expressed in the egg, adult male, and adult female. The RNA tested is indicated on the *x*-axis, and the *y*-axis represents the ratio of *SmInAct* cDNA relative to α-tubulin cDNA (reference gene), as determined by real time RT-PCR. Data are presented as mean ratios (+/− standard deviation [s.d.]) from three separate experiments. There is no significant difference in *SmInAct* expression among the stages tested. (B) SmInAct protein is detectable in eggs, adult males, and adult females from mixed-sex infections in wild-type mice, but is not detectable in females or males from single-sex infections, or in females or males from mixed-sex infections of IL-7R^−/−^ mice. (C) *SmInAct* transcript is localized to the reproductive tissues of the adult female, including the ovary (O) and vitellaria (V) (left panel, in situ hybridization with anti-sense probe) A serial section was probed with sense-strand *SmInAct* and over-developed (right panel). G, gut. Scale bar = 110 μm. (D) *SmInAct* transcript is localized to subtegumental regions of the adult male, with concentrations of expression around the oral sucker (O.S.) and ventral sucker (V.S.) (left panel, in situ hybridization with anti-sense probe). A serial section was probed with sense-strand *SmInAct* and over-developed (right panel). Scale bar = 110 μm. (E) *SmInAct* mRNA levels are significantly lower in females isolated from single-sex infections or from IL-7R^−/−^ mice than in females isolated from infected wild-type mice. *SmInAct* mRNA levels were measured by real-time RT-PCR. Data are presented as mean fold change in expression (+/− s.d.) from two RNA extractions. (F) *SmInAct* mRNA levels in males isolated from single-sex infections or from IL-7R^−/−^ mice compared to mRNA levels in wild-type mice. *SmInAct* mRNA levels were measured by real-time RT-PCR. Data are presented as mean fold change in expression (+/− s.d.) from two RNA extractions. There is no significant difference in *SmInAct* expression in males from single-sex infections or from IL-7R^−/−^ mice versus males isolated from mixed-sex infection of wild-type mice.

To determine the expression of *SmInAct* at the transcript level, real-time reverse transcriptase–polymerase chain reaction (RT-PCR) was performed on cDNA from eggs, adult male parasites, and adult female parasites from mixed-sex infections. As seen in Figure 2A, *SmInAct* is expressed in all stages tested at relatively similar levels. Western analyses using polyclonal antibodies against recombinant SmInAct were used to determine the protein expression profile of SmInAct. The anti-SmInAct serum recognized a 28-kDa protein in egg antigen extracts and a doublet (32 kDa and 28 kDa) in adult male and female extracts (Figure 2B, lanes 1–3); these bands presumably represent the unprocessed (32 kDa) inactive and processed (28 kDa) active forms of the molecule. The relative molecular weights of the two bands recognized by anti-SmInAct antiserum in parasite extracts are larger than that predicted by the sequence, presumably due to detergent and reducing agent-resistant dimerization, and/or to glycosylation at amino acid 110. Glycosylation plays an important role in the solubility and secretion of other members of the TGF-β superfamily [19,20]. Eggs appear to contain only the lower molecular weight, putatively active form of SmInAct. To localize *SmInAct* within the parasite, we performed in situ hybridization on sections of adult worms. Anti-sense probes localized *SmInAct* transcripts to the reproductive tissues of the adult female, with strong signals in the vitellaria and ovary (Figure 2C), whereas in adult males, *SmInAct* transcripts localized to various subtegumental regions (Figure 2D).

The expression pattern in the female suggested a role for *SmInAct* in egg production. We focused on this possibility, and reasoned that if this were the case, *SmInAct* expression might be diminished in unfertile females. In vivo, successful oogenesis requires the presence of male schistosomes [21], and, for reasons that have remained unclear, an intact CD4^+^ T lymphocyte compartment within the host [22]. Therefore, we analyzed *SmInAct* expression in female parasites from mice harboring single-sex infections, and in parasites from severely lymphopenic interleukin-7 receptor knockout (IL-7R^−/−^) mice carrying mixed-sex infections, which produce a significant number of dead eggs [23,24]. Real-time RT-PCR demonstrated that *SmInAct* mRNA levels were significantly decreased, but not absent, in females from these infections (Figure 2E). Of particular interest, SmInAct protein was undetectable by Western analyses in females from single-sex infections as well as from infections of IL-7R^−/−^ mice (Figure 2B).

While the localization of *SmInAct* transcripts to the male subtegumental region is not immediately informative in terms of function in the male, we nevertheless noted that male parasites recovered from infertile infections in IL-7R^−/−^ mice were similar to female parasites in terms of transcriptional and post-transcriptional regulation of SmInAct expression (Figure 2B and 2F). Moreover, this was also the case for male parasites recovered from male single-sex infections (Figure 2B and 2F).

### RNAi-Mediated Knockdown of SmInAct Expression

**Figure 3:**
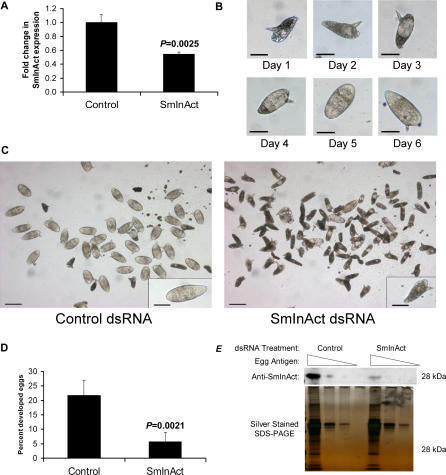
SmInAct Is Essential for Egg Development (A) Treatment of adult parasites with dsRNA corresponding to *SmInAct* led to a 40% reduction in SmInAct mRNA levels. dsRNA treatment is indicated on the *x*-axis, where control worms were treated with luciferase dsRNA. Data are presented as the mean fold change in *SmInAct* expression (+/− s.d.) from three separate experiments, as determined by real-time RT-PCR using paramyosin as a reference gene for expression. (B) Developmental progression of eggs laid in vitro. Eggs produced by paired males and females during the first 48 h *ex vivo* were cultured in vitro for 5 d, and an egg from the developing majority was photographed. Stages of development approximate progressive 20-h periods. Scale bar = 110 μm. (C) Immature eggs produced by adult parasites *ex vivo* and soaked in *SmInAct* dsRNA failed to develop into miracidia. Eggs soaked in an irrelevant control dsRNA (luciferase, 1 μg/ml) developed through stage 6 within 5 d (left) while eggs soaked in *SmInAct* dsRNA (1 μg/ml) for the same period halted development at stage 2 (right). Main scale bar = 210 μm. Inset scale bar = 110 μm. (D) Quantitative analysis of the *SmInAct* dsRNA–induced developmental phenotype. Control- or *SmInAct* dsRNA–treated eggs were examined microscopically and scored as either developed or undeveloped based on the presence or absence of a miracidium. Data are presented as mean percent developed (+/− s.d.) from four separate experiments. (E) SmInAct protein levels are decreased by approximately 10-fold following treatment with *SmInAct* dsRNA. Protein extacts from 350 control or *SmInAct* dsRNA–treated eggs were separated via SDS-PAGE in 10-fold serial dilutions, blotted, and probed with anti-SmInAct antiserum. A silver-stained sister SDS-PAGE gel is shown to confirm protein loading.

To gain a better understanding of the function of SmInAct and the signaling pathway it activates, this TGF-β homolog was targeted for knockdown via RNAi [25–27]. Pairs of adult males and females recovered from infected mice were soaked in double-stranded RNA (dsRNA) corresponding to *SmInAct* (1 μg/ml) or an irrelevant control dsRNA (luciferase) for 1 wk in vitro, followed by RNA extraction and real-time RT-PCR analyses. *SmInAct* dsRNA–treated worms showed a consistent and significant decrease in *SmInAct* expression of >40% when compared to *SmInAct* expression in worms soaked in the irrelevant control dsRNA (Figure 3A). No consistently significant difference in the numbers of eggs produced by control versus *SmInAct* dsRNA–treated worm pairs was observed, suggesting that SmInAct is not important for egg production per se. However, in examining these cultures, we noted that eggs produced by *SmInAct* dsRNA–treated parasites failed to develop (unpublished data). To specifically address the role of SmInAct in egg development, we treated eggs directly with *SmInAct* dsRNA. Approximately 20% of eggs laid by adult parasites during the first 2 d of in vitro culture will develop over the ensuing 5 d to contain miracidia [28], with a typical progression of development through six stages illustrated in Figure 3B. Therefore, eggs produced by worm pairs for the first 2 d *ex vivo* were collected and soaked in dsRNA (1 μg/ml) corresponding to *SmInAct* or an irrelevant dsRNA for 5 d, and their development was scored. Relative to eggs soaked in an irrelevant dsRNA, where ∼20% of the eggs developed through stage 6, eggs treated with *SmInAct* dsRNA aborted development at stage 2 (Figure 3C and 3D). An absence of *SmInAct* transcripts (Figure S1), and a nearly 10-fold decrease in SmInAct protein (Figure 3E), were associated with the failure of *SmInAct* dsRNA–treated eggs to develop. This phenotype was not observed when eggs were treated with dsRNA corresponding to luciferase, a sequence not encoded in the schistosome genome (Figure 3C and 3D), or to S. mansoni cathepsin B1 *(SmCB1),* a cathepsin B detectable in eggs (Table 1).

**Table 1:**
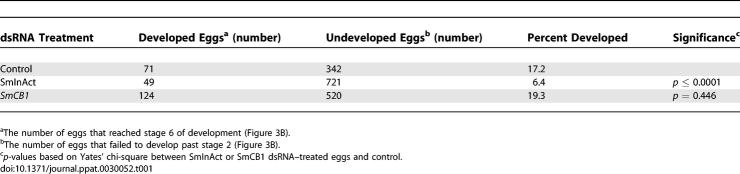
Comparsion of the Development of Eggs Soaked in *SmInAct* dsRNA versus SmCB1 dsRNA

## Discussion

Multiple components of a TGF-β signaling pathway have been characterized in *S. mansoni,* but a ligand of parasite origin for the pathway has remained elusive. Additionally, while functions in host–parasite interactions have been proposed based on the expression of receptors on the parasite surface, and on the responsiveness of the parasite receptors to host TGF-β [5–7,14], the function that TGF-β signaling plays in S. mansoni has remained unclear. In this study, we report the expression of SmInAct, a TGF-β–like ligand in the parasitic flatworm *S. mansoni,* the production of which is coupled to the reproductive potential of the worms. We provide evidence that SmInAct plays a crucial role in embryogenesis.

Understanding of the developmental processes regulated by TGF-β in invertebrates is based largely on data from the model organisms D. melanogaster and C. elegans. *Decapentaplegic,* a bone morphogenetic protein (BMP)–like homolog in *D. melanogaster,* acts as a morphogen by determining cell fate along the dorsal–ventral axis in a gradient-dependent manner [29]. Also in *D. melanogaster,* a type I receptor, *baboon,* stimulates cellular proliferation and is essential for normal embryonic development [30]. Presumably, SmInAct could be fulfilling functions in the schistosome egg analogous to these known roles for decapentaplegic and/or baboon. None of the three characterized TGF-β homologs in C. elegans are important for patterning or growth of the embryo [31–33]; however, two TGF-β homologs have yet to be examined (*tig-2* and Y46E12BL.1), and, intriguingly, serial analysis of gene expression (SAGE) tags for both homologs have been found in the C. elegans embryo [34]. Like the other C. elegans TGF-β homologs that are resistant to RNAi affects, *tig-2* and Y46E12BL.1 have no phenotype in genome-wide RNAi screens [35,36]; therefore, direct mutagenesis will likely be required to determine the function of these genes.

The identification of SmInAct, a TGF-β superfamily member, as a key component of egg development in *S. mansoni,* a member of the Platyhelminthes, the earliest branch of the Bilateria [37], underscores the central role played by this pathway in embryogenesis. While one type I and one type II TGF-β receptor have been characterized for *S. mansoni,* there appears to be at least three type I receptors and two type II receptors present in the genome based on a preliminary blast search for homologs. It will be important to delineate which of the S. mansoni type I and type II TGF-β receptors are involved in SmInAct signaling and to identify the Smads important for transmitting the signal induced by this growth factor. Furthermore, identifying the genes regulated by SmInAct signaling will provide information regarding the precise function that this growth factor serves in egg maturation, as well as the functions the pathway may serve in other life stages of the parasite, including the adult male.

SmInAct protein was not detectable in infertile females recovered from single-sex infections or from IL-7R^−/−^ mice, despite the fact that these parasites contained *SmInAct* transcripts (although at lower levels than in fecund parasites). This strongly indicates that *SmInAct* is both transcriptionally and post-transcriptionally regulated by worms of the opposite sex as well as by signals from the host. It is well established that parasites recovered from hosts lacking CD4^+^ T cells are developmentally stunted and produce significantly fewer fertile eggs than those recovered from mixed-sex infections of immunocompetent hosts. Translation of SmInAct mRNA is the first identified molecular process downstream of the effect of the host immune system on schistosome development [22–24], and as such, could open the way towards an increased understanding of this unusual feature of schistosome biology. The finding that the production of SmInAct in males is under the same constraints as in females is curious and perhaps indicates an additional function(s) for SmInAct in S. mansoni. We are unaware of a link between the site of expression of SmInAct in the male schistosome and reproductive events, and further work is required to elucidate the function of SmInAct in male worms.

In other settings, the uncoupling of transcription and translation is linked to the activation of the integrated stress response [38–41]. This mechanism, conserved in eukaryotes, re-programs cells to conserve energy in response to stress signals such as amino acid deficiency and oxidative stress by restricting the translation of transcripts requiring an active translation initiation complex [38–41]. Limited cellular energy is then used for the expression of genes necessary to maintain cell viability [42]. In this context, parasites in single-sex infections and in mice lacking CD4^+^ T cells may be considered stressed due to the lack of signals received from the opposite sex and immunocompetent host, thereby restricting the translation of non-essential transcripts. SmInAct protein expression may be considered expendable considering the role it plays in embryogenesis rather than in crucial cellular functions linked to the survival of the adult worm. A more thorough investigation of the S. mansoni homologs of translation factors involved in the stress response and of the regulation of other transcripts and protein expression will be required to evaluate this possibility.

Post-transcription regulation of eukaryotic transcripts is controlled in part by the 3′UTR [43]. This region can bind elements (including microRNAs and proteins) that inhibit the translation and/or decrease mRNA stability. For example, 3′UTRs of several mammalian cytokines contain adenosine- and uridine-rich elements (AREs) that bind ARE-binding proteins (ARE-BPs) (reviewed in [44]). The binding of ARE-BPs to these transcripts causes either rapid decay or inhibits their translation. While AREs are somewhat divergent in sequence, they often contain the consensus “AUUUA” and are found in a uridine-rich environment. Interestingly, the long 3′UTR of *SmInAct* has two exact repeats of “UUUCTAUUUA” that contain the consensus “AUUUA” ARE (underlined). Furthermore, the 3′UTR of *SmInAct* is U-rich (43% uridines). It will be interesting to determine whether these repeats, or other regions of the long 3′UTR, play a role in the post-transcriptional regulation of *SmInAct* expression.

It is of interest when considering the relationship of schistosomes with their mammalian hosts to note that in other systems, TGF-β superfamily members have been shown to function across phylum boundaries [45,46]. For example, the *Drosophila* BMP homologs DPP and 60A are able to induce bone development when injected into the skin of rats [45], and mammalian BMP-4 can rescue *Drosophila* DPP mutants [46]. Consequently, we believe that it is feasible that SmInAct could act as a ligand to initiate signaling in host cells. It is clear that proteins produced by eggs have distinct immunomodulatory functions [47], and SmInAct could conceivably participate in these effects if secreted/excreted from the schistosome egg. Our identification of SmInAct as a cytokine that is molecularly conserved between host and parasite, coupled with the description of an effective method for altering gene expression in the schistosome egg, allows these and other issues to now be addressed. Despite recent advances in vaccine design [48], a solution for schistosomiasis remains an elusive goal. Current attempts to control schistosomiasis depend on repeated administration of one drug, praziquantel, with no replacements waiting in the wings should resistance develop. Understanding how schistosome eggs develop could provide targets for intervention in the schistosome life cycle and for blocking disease progression.

## Materials and Methods

### Parasites and animals.

The Puerto Rican/NMRI strain of S. mansoni was used in all experiments. Adult schistosomes were recovered by hepatic-portal perfusion from C57BL/6 female mice or B6 IL-7R^−/−^ (The Jackson Laboratory, http://www.jax.org) that had each been percutaneously exposed to ∼60 cercariae 8 wk earlier. Adult parasites and eggs laid were maintained in vitro in M199 (Gibco, http://www.invitrogen.com), 10% fetal calf serum, 1% Antibiotic/Antimycotic (Gibco), and 1% HEPES in a 37 °C/5% CO_2_ atmosphere as previously described [11,28].

### Isolation of full-length *SmInAct* cDNA from *S. mansoni.*

The C-terminal, translated region of the *Drosophila* activin homolog (dActivin) (amino acids 565–669) was used to search the Wellcome Trust's Sanger Institute's S. mansoni genome assembly using the t*blast*n algorithm. A contig (0020320) with significant similarity to dActivin was identified. Full-length cDNA corresponding to *SmInAct* was isolated using total RNA (1 μg) from adult parasites and the SuperScript III GeneRacer 5′ and 3′ RACE kit (Invitrogen, http://www.invitrogen.com) as per manufacturer's instructions. Gene-specific primers were designed for isolation of the 5′-end (5′-GGTTCAAAACTTTTCGGGTGTA-3′) and 3′-end (5′-AATCTTGTTGTCATCCAACTCAA-3′) of *SmInAct* and used in RT-PCR with GeneRacer 5′ and 3′ primers according to manufacturer's suggestions. Resulting amplicons were cloned into the TOPO cloning vector (Invitrogen) and sequenced. To verify the full-length sequence of *SmInAct,* primers designed from the 5′ and 3′ ends of the transcript were used in RT-PCR, and the resulting fragment was cloned and sequenced.

### Sequence analysis.

Sequence similarities between the deduced amino acid sequence of SmInAct and other members of the TGF-β superfamily were determined through multiple sequence alignments using the ClustalW algorithm, as well as the Align 2 sequences (bl2seq) program at the National Center for Biotechnology Information (http://www.ncbi.nlm.nih.gov). An unrooted phylogram was drawn using amino acids within the conserved C-terminal domain of SmInAct, and known TGF-β superfamily members and distances were drawn using the Dayhoff Pam matrix and neighbor-joining algorithm in the PHYLIP software package developed by J. Felsenstein, University of Washington, Seattle, Washington, United States (http://evolution.genetics.washington.edu/phylip.html). Percentages at branch points are based on 1,000 bootstrap runs.

### Real-time RT-PCR.

Total RNA was extracted from parasites using Qiagen's RNeasy Mini kit (http://www.qiagen.com), and contaminating genomic DNA was removed by DNase treatment using the Turbo DNA-free endonuclease (Ambion, http://www.ambion.com). First-strand cDNA was synthesized using 500 ng of RNA, SuperScript II reverse transcriptase (Invitrogen), and oligo dT as a primer. RT-minus controls were performed to confirm the absence of genomic DNA (unpublished data).

SmInAct transcript levels in egg and adult stages were quantified relative to α-tubulin using Applied Biosystems' 7500 real-time PCR system and SYBR green PCR Master Mix (Applied Biosystems, http://www.appliedbiosystems.com). Total reaction volume was 10 μl with 300 nM of each primer, 5 μl of SYBR green PCR Master Mix, and 0.5 μl of cDNA as template (or water as a negative control). SmInAct primers were: forward 5′-AATCTTGTTGTCATCCAACTCAA-3′ and reverse 5′-AACTACAAGCACATCCTAAAACAA-3′. α-Tubulin primers were: forward 5′-CCAGCAAATCAGATGGTGAA-3′ and reverse 5′-TTGACATCCTTGGGGACAAC-3′. PCR efficiency (E) was determined for both primer sets by plotting cycle thresholds from a 10-fold serial dilution of cDNA and inputting the slope in the equation E = 10^(−1/slope)^. For expression analyses, quantification of SmInAct transcript relative to α-tubulin was calculated using the equation: ratio = (*E*_Smα-tubulin_)^CT^/(*E_SmInAct_*)^CT^ where *E*_Smα-tubulin_ is the PCR efficiency of the reference gene, *E_SmInAct_* is the PCR efficiency of target gene, and CT is the cycle threshold. For analysis of RNAi-induced knockdown, quantification of SmInAct transcript relative to paramyosin (paramyosin primers were: forward 5′-CGTGAAGGTCGTCGTATGGT-3′ and reverse 5′-GACGTTCAAATTTACGTGCTTG-3′) was calculated using the 2^−ΔΔCt^ method. Dissociation curves were generated for each real-time RT-PCR to verify the amplification of only one product.

### Recombinant SmInAct expression, antiserum production, and Western analyses.

Eco RI (forward) and Xho I (reverse) tagged primers were designed to amplify the C-terminal bioactive region of SmInAct (forward 5′-GGAATTCTCATTAACTAAAGGAGATGA-3 and reverse 5′-CCGCTCGAGTTAACTACAAGCACATCCTA-3′). The amplified product was cloned into the expression vector pET28a+ (Novagen, http://www.emdbiosciences.com) and sequenced to verify the absence of any mutations. Expression of recombinant SmInAct was induced in Escherichia coli BL21(DE3) by addition of 1 mM IPTG when cultures reached an OD_600_ of 0.5 at 37 °C, followed by 3 hours of shaking at room temperature. Recombinant SmInAct was expressed in bacteria as insoluble inclusion bodies. Exhaustive attempts to refold the protein using gluathione and reduced glutathione proved unsuccessful. We therefore purified the protein via nickel column chromatography under denaturing conditions (6 M urea) as per the manufacturer's protocol (Novagen). Antiserum was generated by Cocalico Biologicals (http://www.cocalicobiologicals.com) through subcutaneous inoculation of a rabbit with 100 μg of purified protein in complete Freund's adjuvant, followed by three boosts of 50 μg in incomplete Freund's adjuvant on days 14, 21, and 49, followed by exsanguinations on day 64.

For detection of SmInAct protein, 10 μg of protein extracted from eggs, adult males, and adult females via Dounce homogenizing in lysis buffer (1% Triton-X 100, 20 mM HEPES, 10% glycerol, 150 mM NaCl) supplemented with a protease inhibitor cocktail (Sigma, http://www.sigmaaldrich.com) were separated by SDS-PAGE, electroblotted, and probed with anti-SmInAct antiserum (1:10,000), pre-immune serum (1:10,000), or a monoclonal antibody (4B1) against paramyosin. Affinity purified HRP-conjugated goat anti-rabbit IgG (Cell Signaling Technology, http://www.cellsignal.com) was used to detect bound rabbit antibodies, while an affinity purified HRP-conjugated horse anti-mouse IgG (Cell Signaling Technology) was used to detect the anti-paramyosin monoclonal antibody. The secondary antibodies were detected using ECL reagents as per manufacturer's instructions (GE Healthcare, http://www.gehealthcare.com).

### In situ hybridization.

Localization of SmInAct in 5-μm sections of adult S. mansoni was performed as previously described [49]. DIG-labeled sense and anti-sense transcripts were generated using Roche's DIG RNA labeling mix (http://www.roche.com) as per manufacturer's instructions with T7-tagged amplicons as template (sense: forward 5′-TAATACGACTCACTATAGGGTTGATCCAAAAAAGGTTGTTATGG-3′, reverse 5′-TTAACTACAAGCAGCTCCTA −3′; anti-sense: forward 5′-ATAATATGTAATAATTGTGA −3′ reverse 5′- TAATACGACTCACTATAGGGAACTACAAGCACATCCTAAAACAA-3′). The hybridized DIG-probes were detected using an alkaline-phosphatase conjugated anti-DIG antibody (Roche), and visualized using NBT (337.5 μg/ml) and BCIP (175 μg/ml) in 0.1M Tris-HCl, 0.1M NaCl, 0.05 MgCl_2_. Worm sections were photographed using a Leica DMIRB microscope and DC500 camera (Leica, http://www.leica.com).

### dsRNA synthesis and RNA interference.

dsRNA was synthesized using the T7 Megascript kit (Ambion) as per manufacturer's instructions. T7-tagged primers were used to generate a 381-bp *SmInAct*-dsRNA template encompassing the active ligand domain (forward 5′-TAATACGACTCACTATAGGGCGATCATTAACTAAAGGAGATGAG-3′, reverse 5′-TAATACGACTCACTATAGGGAACTACAAGCACATCCTAAAACAA-3′). Luciferase and SmCB1 dsRNAs (negative controls) were generated as described [25]. For dsRNA treatment of worms, five adult pairs were cultured in the presence of 1 μg/ml dsRNA for 7 d with medium and dsRNA changes occurring every other day. For dsRNA treatment of eggs, five adult pairs were cultured as above (in the absence of dsRNA) for 2 d, worms were removed, and dsRNA was added at 1 μg/ml. Eggs were photographed using a Leica DMIRB microscope and DC500 camera.

### Statistical analyses.

Student *t*-test was used for statistical analyses of dsRNA-induced knockdown of SmInAct expression, change in expression of SmInAct in single-sex and IL-7R^−/−^ mice, and egg developmental phenotypes (control versus *SmInAct* dsRNA). Chi-square analyses were used to test the statistical significance of the egg developmental phenotype. The Yates correction was applied because we specified only two categories: undeveloped and developed (Table 1).

## Supporting Information

Figure S1 *SmInAct* Transcript Is Not Detectable in *SmInAct* dsRNA–Treated Eggs *SmInAct* mRNA levels in eggs treated with *SmInAct* dsRNA or control dsRNA for 5 d were measured using real time RT-PCR using paramyosin as a reference gene for expression. dsRNA treatment is indicated on the *x*-axis. Data are presented as the mean fold change in SmInAct expression. N/D = not detected. In this experiment, paramyosin mRNA was detectable in the *SmInAct* dsRNA–treated eggs. However, in most experiments in which eggs were treated with *SmInAct* dsRNA, it was not possible to recover mRNA from which reference transcripts could be detected by RT-PCR. Eggs treated with control dsRNA always yielded high quality mRNA.
(560 KB TIF)

### Accession Numbers

Sequence data reported in this manuscript are available from GenBank (http://www.ncbi.nlm.nih.gov/Genbank) under accession number DQ863513. Other GenBank accession numbers of genes and sequences used in this study include: B. malayi TGH-1 (AAB71839); B. malayi TGH-2 (AAD19903); C. elegans DAF-7 (NP_497265); C. elegans DBL-1 (NP_504709); Danio rerio Activinβ A isoform 2 (AAX68505); D. melanogaster Activin (NP_651942); D. melanogaster dActivin (AF454392); *D. melanogaster* decapentaplegic (NP_477311); Homo sapiens Activinβ E (NP_113667); H. sapiens BMP-2 (NP_001191); H. sapiens BMP-3 (NP_001192); H. sapiens BMP-4 (NP_031580); H. sapiens BMP-5 (NP_066551); H. sapiens BMP-6 (NP_001709); H. sapiens BMP-7 (NP_001710); H. sapiens BMP-8 (NP_861525); H. sapiens GDF-5 (NP_000548); H. sapiens GDF-6 (NP_001001557); H. sapiens GDF-7 (NP_878248); H. sapiens GDF-10 (NP_004953); H. sapiens Inhibinβ A precursor (NP_002183); H. sapiens Inhibinβ B (NP_002184); H. sapiens Inhibinβ C (NP_005529); H. sapiens TGF-β 1 (NP_000651); H. sapiens TGF-β 2 (NP_003229); H. sapiens TGF-β 3 (NP_003230); Mus musculus BMP-2 (NP_031579); M. musculus BMP-3 (NP_775580); M. musculus BMP-4 (NP_031580); M. musculus GDF-10 (NP_665684); M. musculus Inhibinβ A (NP_032406); M. musculus Inhibinβ B (NP_032407); M. musculus TGF-β 1 (NP_035707); M. musculus TGF-β 2 (NP_033393); M. musculus TGF-β 3 (NP_033394); S. mansoni α-tubulin (M80214); S. mansoni paramyosin (M35499); and Strongyloides stercoralis TGH-1 (AAV84743).

## Abbreviations

ARE: adenosine- and uridine-rich element
ARE-BP: adenosine- and uridine-rich element–binding protein
bp: base pairs
BMP: bone morphogenetic protein
dsRNA: double-stranded RNA
IL-7R^−/−^: interleukin-7 receptor knockout
RNAi: RNA interference
RT-PCR: reverse transcriptase–polymerase chain reaction
SmCB1: *S. mansoni* cathepsin B1
SmInAct: *S.* *mansoni* Inhibin/Activin
s.d.: standard deviation
TGF-β: transforming growth factor–β
UTR: untranslated region
